# Multi-proxy record of the Austrian Upper Triassic Polzberg *Konservat-Lagerstätte* in light of the Carnian Pluvial Episode

**DOI:** 10.1038/s41598-024-60591-9

**Published:** 2024-05-21

**Authors:** Alexander Lukeneder, Petra Lukeneder, Reinhard F. Sachsenhofer, Guido Roghi, Manuel Rigo

**Affiliations:** 1 Geological-Paleontological Department, Natural History Museum Vienna, Burgring 7, 1010 Vienna, Austria; 2 Vienna Doctoral School of Ecology and Evolution, University of Vienna, Djerassiplatz 1, 1030 Vienna, Austria; 3 Department Applied Geosciences and Geophysics; Montanuniversität, 8700 Leoben, Austria; 4 Institute of Geosciences and Earth Resources, IG, National Research Council CNR, Via G. Gradenigo 6, 35131 Padua, Italy; 5 Department of Geosciences, University of Padua, Via G. Gradenigo 6, 35131 Padua, Italy

**Keywords:** Environmental sciences, Solid Earth sciences

## Abstract

We present a multi-proxy investigation of a lower Carnian basinal succession from Polzberg in the Northern Calcareous Alps (Lower Austria). A section comprising a unique *Konservat-Lagerstätte* was studied based on bio- and chemostratigraphy along with geophysical methods, yielding a detailed and robust stratigraphic calibration of the Polzberg succession. The Polzberg section revealed the paleoceanographic history and helped to identify a global climatic reversal, the Carnian Pluvial Episode. The age of the Upper Triassic Reingraben formation in the Northern Calcareous Alps is refined as the *Austrotrachyceras austriacum* Zone within the lower Carnian (Julian 2). Ammonoids and conodonts provide a detailed biostratigraphic subdivision that serves as a basis for analyses of the faunal distribution and the paleoenvironmental evolution of the Upper Triassic Reifling Basin. The succession includes lithological and facies changes similar to those of coeval units in the Tethys. The Carnian was characterized by a weak (~ 1‰) positive δ^13^C trend, punctuated by a negative shift during the lower Carnian corresponding to the initiation of the Carnian Pluvial Episode, a period representing the onset of early/late Carnian transitional global greenhouse conditions. Organic maturity parameters and the conodont alteration index (CAI) show that the thermal overprint of the Polzberg section is low. Biomarker proxies suggest that the organic matter of the uppermost Göstling formation is a mixture of marine and terrestrial material deposited in a dysoxic environment. Within the overlaying Reingraben formation, the amount of marine biomass decreased gradually upwards. Oxygen-depleted conditions, probably due to water-column stratification, continued during deposition of the Reingraben formation. Bacterial sulfate reduction played a major role in organic matter degradation.

## Introduction

During the Late Triassic an important global climatic episode took place, referred to as the Carnian Pluvial Episode (CPE^1–5^), Carnian Pluvial Event (CPE^6,7^), Carnian Pluvial Phase (CPP^8^) or Carnian Crisis (CC^9–11^). Depositional records and sections, especially from localities around the western Tethys, documented a climatic transition to warmer and more humid conditions^1,2,12,13^. These climatic perturbations were mainly related to an increase in the volcanic activity at the Wrangellia large igneous province^14,15^. This triggered episodes of climate warming, enhanced rainfall and freshwater influx into the marine basins, as well as increased burial of organic carbon and humidification^1–5,8^. The evidence shows that during the early Carnian (Julian), global temperatures started to rise, shifting the system towards warm and humid climatic conditions ca. 2 million years. These conditions peaked at the Julian/Tuvalian boundary (lower Carnian/upper Carnian boundary^12,16,17^).

**Figure 1:**
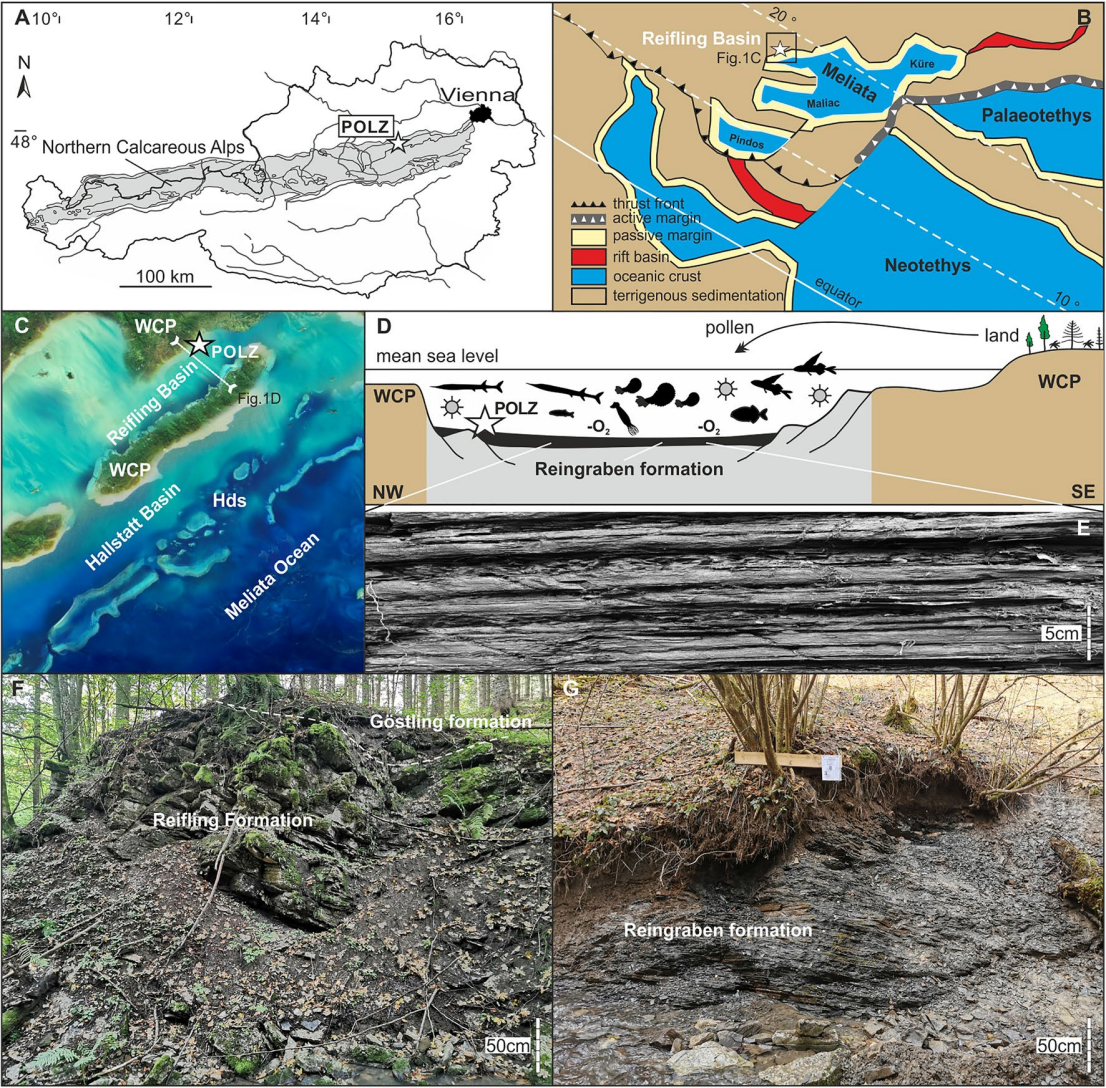
Locality map of the Upper Triassic Polzberg area. (**A**) Polzberg area (white star) within the Northern Calcareous Alps (NCA, Lower Austria). (**B**) Paleogeographic position of the Polzberg Reifling Basin with indicated outcrop position in (**C**). (**C**) Palinspastic position of the Reifling Basin and the Polzberg locality with indicated transverse section (**D**). (**D**) Transect through the Reifling Basin with reconstruction of basinal environments low oxygen conditions (–O_2_) and surrounding land with plants. (**E**) Laminated character of the Reingraben Shales at the Polzberg *Konservat-Lagerstätte*. (**F**) Outcrop situation at Polzberg, Reifling Formation and basal Göstling formation. Unit boundary marked by a white dashed line. (**G**) Basal Reingraben formation at Polzberg. (**F**, **G**) images by AL April 2023. White asterisks: position of the Polzberg *Konservat-Lagerstätte* (POLZ). WCP, Wetterstein Carbonate Platform; Hds, Hallstatt deepwater swells. NCA area in gray. (**B**–**D**) not to scale. Adapted after Lukeneder & Lukeneder^4^. (**B**) adapted after Lukeneder et al.^27^. Artwork of map in (**C**) by Mathias Harzhauser, NHMW. Prepared by AL using CorelDraw X7; www.coreldraw.com.

The Upper Triassic Polzberg section in Austria (Fig. 1) comprises a unique *Konservat-Lagerstätte*^18,19^ and is one of the best reference sections for the CPE in Austria and in the world because the biostratigraphy and taxonomy, taphonomy and trophic relations of various marine organisms of the Reifling Basin^4,20,21^ are well studied. This section therefore represents a key section for the lower Carnian *Konservat-Lagerstätte* worldwide, making it an Upper Triassic high-quality depositional archive of marine ecosystems for this period. Known taxa from historical and recent fossil collections of the Polzberg area including invertebrates, vertebrates, trace fossils and plants reveal new details of the Carnian food webs from this unique *Konservat-Lagerstätte*.

The Carnian paleobiota from the Polzberg *Konservat-Lagerstätte*^4,22,23^ has been historically and recently studied for several topics, yielding results on the paleoenvironment and trophic chain^4,24^ as well as on the taxonomy and taphonomy of ammonoids^20,25^, including belemnoid cartilage investigations^21^.

**Figure 2:**
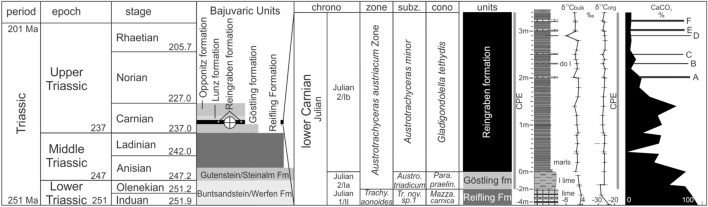
Stratigraphic position of the lower Carnian Reingraben formation at Polzberg with the layers comprising the Upper Triassic (lower Carnian) Polzberg paleobiota within the *Austrotrachyceras austriacum* Zone. Log with indicated lithological units, thickness of the Polzberg section, curves on δ^13^C_bulk rock_ and δ^13^C_org_ (detailed curves in Fig. 7), CaCO_3_ content and calcareous dolostone-layers (**A**–**F**). CPE, Carnian Pluvial Episode with vertical gray bars. chrono, chronostratigraphy; cono, conodonts; l lime, laminated limestones; lime, limestones; sunz. subzone; *Para. praelin.*, *Paragondolella praelinda*; *Mazza.*, *Mazzaella*; zone and subzone correspond to ammonoid taxa. Prepared by AL using CorelDraw X7; www.coreldraw.com.

Although discovered more than 140 years ago, the formation and depositional processes of the lower Carnian Polzberg *Konservat-Lagerstätte* are still not well understood. Four field campaigns between 2021 and 2022 enabled collecting fossils and rock samples bed-by-bed, and detailed investigations of the fauna and flora here yielded new insights into the CPE paleocommunity^4,20,21,24,26^. A detailed geochemical examination, however, is still lacking. Deposited during the Carnian Julian 2 Ib (*Austrotrachyceras austriacum* Zone, *A. minor* Subzone; Fig. 2), the Polzberg *Konservat-Lagerstätte* (PK-L) depicts a short but precise fingerprint of the CPE^4,27,28^. The deposition of the fossiliferous Reingraben formation at Polzberg, reflecting the severe environmental change during the CPE, features increasingly humid conditions during the early Carnian. This enhanced the preservation of entire and soft body fossils, which is a prerequisite for and typical of *Konservat-Lagerstätten*. The paleobiota reflects a normal marine environment, deposited in a laminated and low-oxygenated environment with intermittent shallow-water influence^4,29–31^.

In order to establish the Polzberg deposits as a reference section (Polzberg, Po) for the lower Carnian in the western Tethyan region, we present here a more detailed age model of the lower Carnian Polzberg deposits. It is based on detailed macro- and microfossil biostratigraphic data within a geochemical and geophysical framework. Carbon (δ^13^C_bulk_, δ^13^C_org_), CaCO_3_, total organic carbon (TOC), total sulfur content (TS), organic matter (OM), biomarker (*n*-alkanes, steroids, hopanoids), the Color Alteration Index (CAI) of conodonts, geochemical proxies combined with magnetic susceptibility (MS) and gamma-ray measurements (GR) have been investigated and are here presented to describe the environmental evolution of the Reifling Basin.

### Geological setting

The Upper Triassic outcrops at Polzberg are located on the western slope of Mount Schindelberg (1066 m), north of the Ois River, 4 km northeast of Lunz am See in Lower Austria. The locality Schindelberg is synonymous with the locality Polzberg (= Pölzberg^4,29,30^; 1:50,000, geological map, sheet 71 Ybbsitz^32^, and sheet 72 Mariazell^33^; Fig. 1). The northernmost tectonic elements of the Northern Calcareous Alps (NCA, Bajuvaric Units) in Lower Austria are the Frankenfels Nappe, followed to the south by the Lunz Nappe. Within the Lunz Nappe, the Reifling Basin^4,22^—an Upper Triassic intraplatform basin—is located between Polzberg and Großreifling. The exact position of the fossiliferous localities in the southern area of the Lunz Nappe within the lower, fossiliferous part of the Reingraben formation^34,35^ was determined by GPS (global positioning system): N 47° 53′ 4.98″ and E 15° 4′ 28.15″, town Gaming, federal district Scheibbs.

### Multi-proxy data on the Polzberg *Konservat-Lagerstätte*

The lithologies at Polzberg can be subdivided into three main lithostratigraphic units that, in stratigraphic order, are the Reifling Formation (formalized), the Göstling formation (= Göstlinger Kalke; not formalized; frequently noted as Göstling formation of the uppermost part of the Reifling Formation) and the Reingraben formation (= Reingraben Shales, Reingrabener Schiefer; not formalized; Fig. 2). The main lithological units are here understood as well-definable formations, but not yet formalized herein.

#### Lithology and microfacies

**Figure 3:**
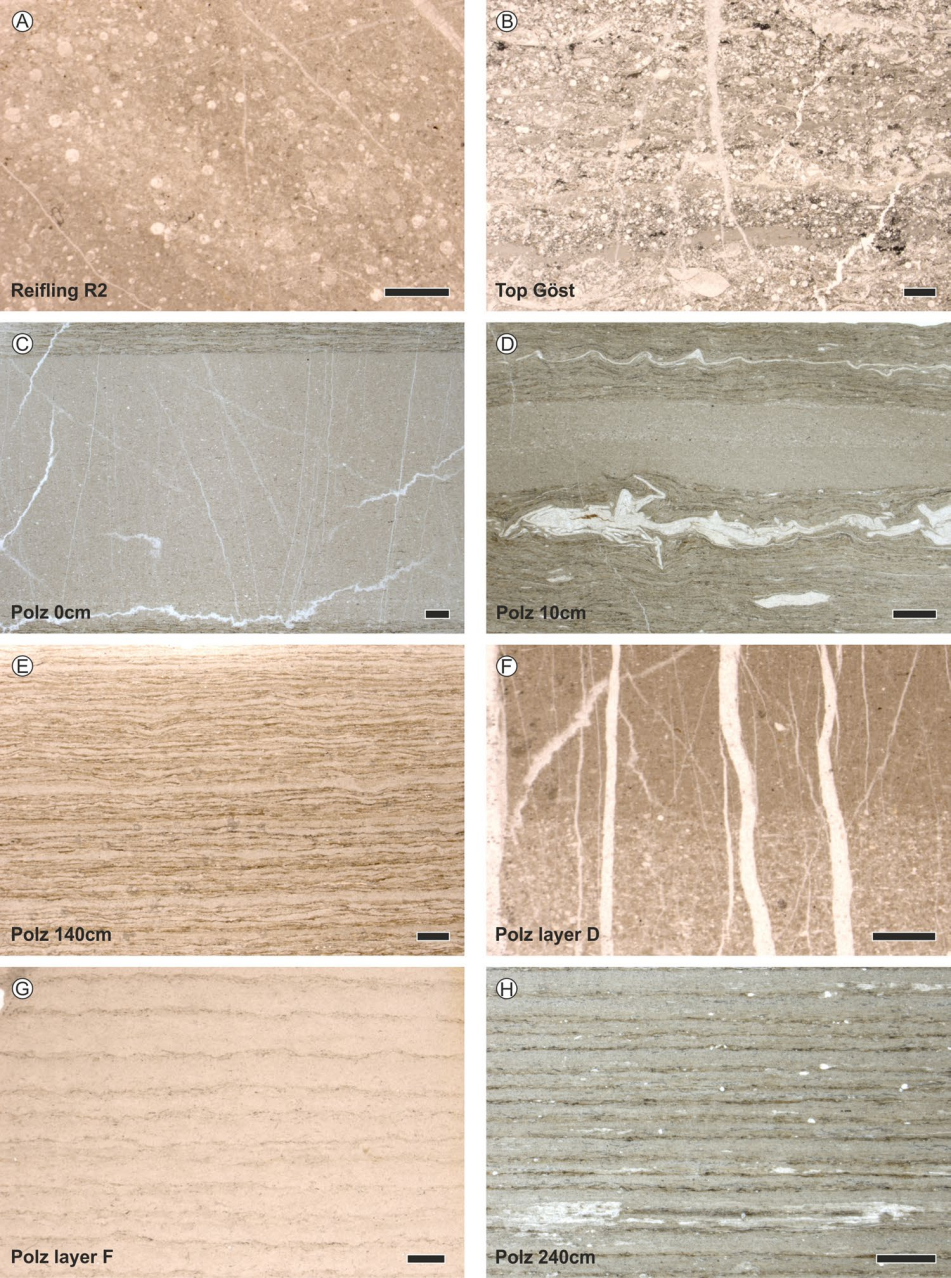
Thin sections from the basal Reifling Formation, the Göstling formation and the Reingraben formation form the Polzberg deposits. (**A**) Radiolarian-rich limestone (wackestone) of the middle part of the Reifling Formation, Reifling R2. (**B**) Thin layered cherty limestones (wackestone-packstone), biogenous limestone wirth dominant radiolaria and bivalves of the Göstling formation, Top Göst. (**C**) Intercalated calcareous layer (dolostone) within the basal Reingraben formation, Polz 0 cm. (**D**) Basal laminated more argillaceous layers of the Reingraben formation, with whitish translucent ammonoid shell remnants, Po 10 cm. (**E**) Fine laminated layers of the middle part of the Reingraben formation, Po 140 cm. (**F**) Thin calcareous calciturbidite layer transformed to dolostone, with foraminifera remnants, Po layer D. (**G**) Calcareous dolostone-layer with visible traces of primary lamination, Po layer F. (**H**) Fine laminated Reingraben formation with abundant gastropod remnants, Po 240 cm. Normal transmitted light. Polz, Polzberg; Top Göst, Top Göstling formation. Scale bars 1 mm. Prepared by AL using CorelDraw X7; www.coreldraw.com.

**Figure 4:**
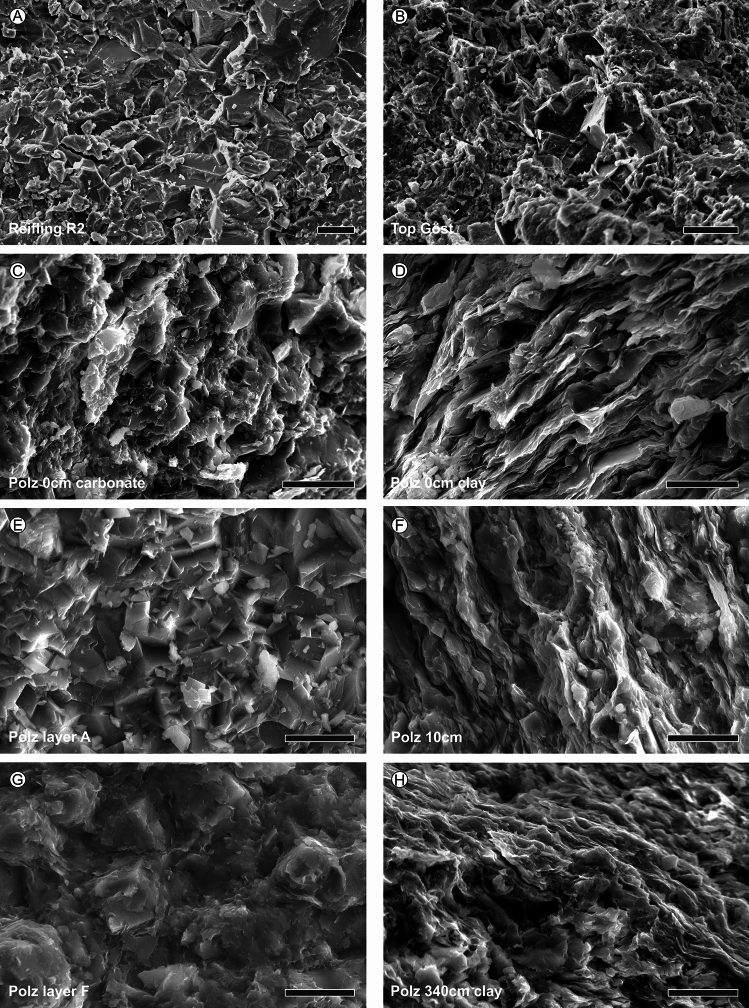
SEM images from different fresh lithological surfaces of the basal Reifling Formation, the Göstling formation and the Reingraben formation form the Polzberg deposits. (**A**) Limestone with angular broken surface of the middle part of the Reifling Formation, Reifling R2. (**B**) Limestones wirth cherty angular area in the center of the Göstling formation, Top Göst. (**C**) Intercalated calcareous layer with angular dolomite crystals within rare clay flakes in the basal Reingraben formation, Polz 0 cm carbonate. (**D**) Wavy laminated argillaceous layers with domoinant clay flakes in the basal Reingraben formation, Po 10 cm clay. (**E**) Calcareous dolostone layer with anular dolomite crystals in the middle part of the Reingraben formation, Po layer A. (**F**) Claystone layer with angular dolomite crystals swimming in the clayey matrix within the basel Reingraben formation, Po 10 cm. (**G**) Calcareous dolostone-layer with angular dolomite crystals covered by clay flakes located in the upper part of the Reingraben formation, Po layer F. (**H**) Fine wavy laminated claystones of the upper Reingraben formation, Po 340 cm. Polz, Polzberg; Top Göst, Top Göstling formation. Scale bars 10 µm. Prepared by AL using CorelDraw X7; www.coreldraw.com.

The Reifling Formation (− 4 to − 2 m, beds R1 and R2) was deposited on the paleoslope of the Reifling Basin^4^. It consists of light grey and grey-green nodular limestones composed of peloidal, filamentous wackestone and packstones (Figs. 3, 4). Bed-thickness (0.2–0.4 m) and carbonate content are stable throughout the formation. Rare fish and marine reptile remnants are visible.

At the base of Julian 2, the Reifling Formation (*Trachyceras aonoides* Zone, *T. aonoides* Subzone) was replaced by the Göstling formation (− 2 to 0 m, R3 and Göst top), which represents the *Austrotrachyceras austriacum* Zone (*A. minor* Subzone). The Göstling formation consists of black, mm- to cm-bedded calciturbiditic radiolarian limestones with organic-rich mudstones. The change from the Reifling Formation to dark, fine-laminated radiolaria wackestones to packstones of the Göstling formation is abrupt. At Polzberg, only about 2 m of radiolarian-rich Göstling limestones can be observed, whose thickness may have been reduced by tectonics.

The overlaying is in introduction terrigenous siliciclastic deposits of the Reingraben formation (0 to 340 cm, Po 0 cm to Po 340 cm; *A. austriacum* Zone, *A. minor* Subzone) consist of a pronounced alternation of dark grey to black argillaceous marls intercalated by rare limestone/dolomite layers (layer A–F); no macro-bioturbation is visible (Figs. 3, 4).

Microfacies consist mainly of biomicritic limestones, often slightly bioturbated, differentiated into foraminiferal- to radiolarian-dominated wackestones to packstones. The lower Carnian interval is generally abundant in microfossils. Calcified radiolarians and sponge spicules are abundant in the packstones of both formations, while ammonoid and bivalve mudstones appear from the base of the Reingraben formation. A sudden change from radiolarian wackestones/packstones of the Göstling formation occurs above the boundary to the Reingraben formation.

#### Ammonoid biostratigraphy

No ammonoids are present in the Reifling Formation, and only rare trachyceratid specimens (*Austrotrachyceras patroclus*) are present in the Göstling formation. *Austrotrachyceras patroclus* is characteristic for the basal *Austrotrachyceras austriacum* Zone (*A. minor* Subzone). As described in previous papers^4,20,25^, the lowermost, fossiliferous part of the Reingraben formation can be dated based on the occurrence of *Austrotrachyceras minor*, *Paratrachyceras haberfellneri* and the accompanying *Carnites floridus* and *Simonyceras simonyi*, indicating a Julian 2 age (Julian 2/Ib; Fig. 2). The presence of these ammonoid members together with the mass occurrence of the benthic bivalve *Halobia rugosa* within the fossiliferous layers confirms the biostratigraphical age assignment as Julian^4,8,20,21,25^.

#### Conodont biostratigraphy

**Figure 5:**
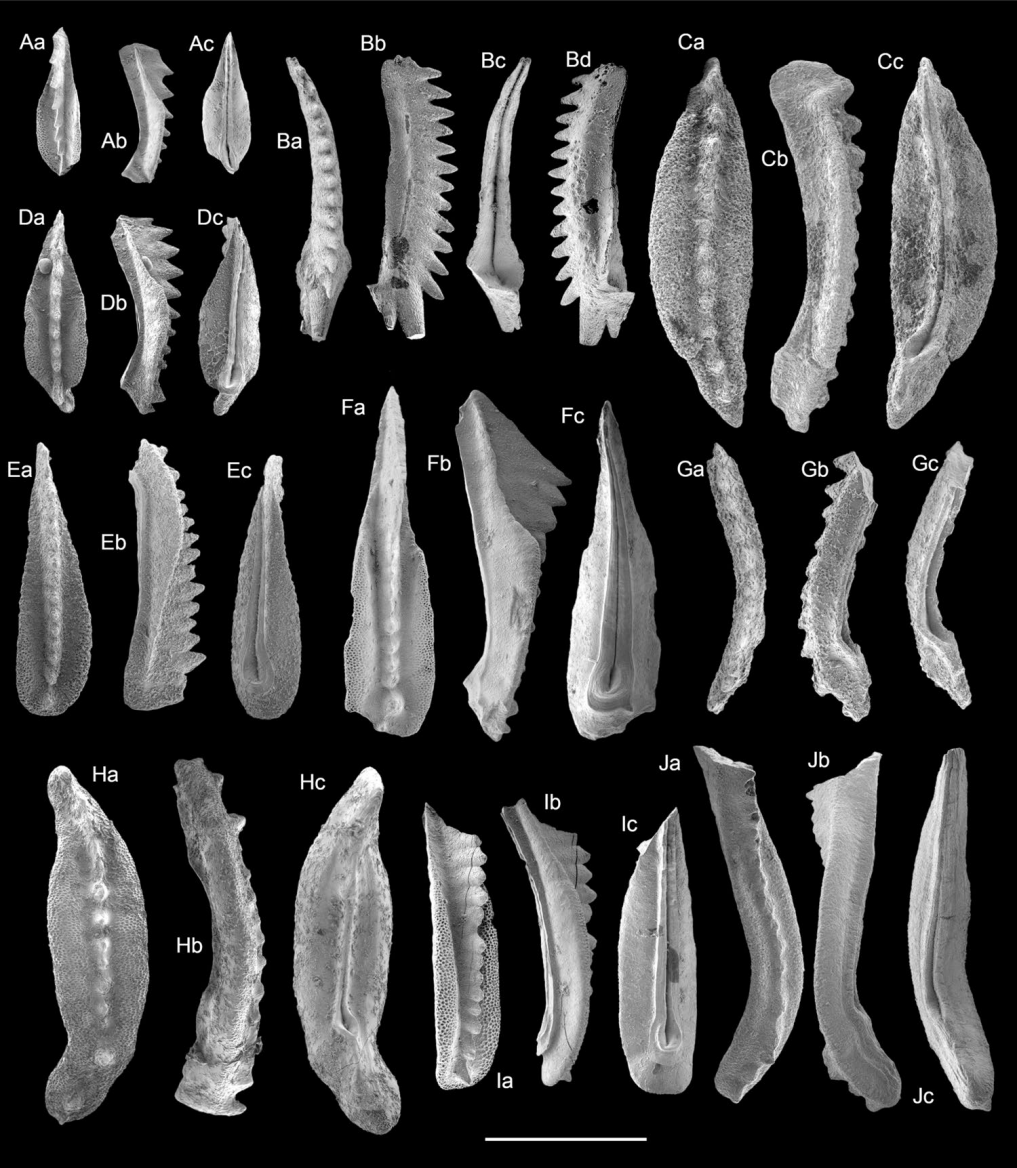
SEM micrographs of conodonts from Polzberg section. (**A**) *Paragondolella* aff. *Par. foliata* (sensu Rigo et al.^6^), sample Top Reif Polz 0.063, NHMW 2021/0123/0517. (**B**) *“Mosherella" longnanensis,* sample Top Reif Polz 0.125 NHMW 2021/0123/0518. (**C**) *Gladigondolella malayensis malayensis*, sample Top Reif Polz 0.125, NHMW 2021/0123/0519. (**D**) *Paragondolella praelindae*, sample Top Reif Polz 0.063, NHMW 2021/0123/0520. (**E**) *Paragondolella foliata*, sample Top Reif Polz 0.125, NHMW 2021/0123/0521. (**F**) *Paragondolella polygnathiformis*, sample Top Reif Polz 0.25, NHMW 2021/0123/0522. (**G**) *Gladigondolella arcuata*, sample Top Reif Polz 0.125, NHMW 2021/0123/0523. (**H**) *Gladigondolella malayensis malayensis*, sample Base Göst Polz 0.25, NHMW 2021/0123/0524. (**I**) *Paragondolella inclinata*, sample Top Reif Polz 0.125, NHMW 2021/0123/0525. (**J**) *Gladigondolella tethydis*, sample Top Reif Polz 0.25, NHMW 2021/0123/0526. Scale bar 400 µm. Prepared by MR using CorelDraw X7; www.coreldraw.com.

Conodonts are restricted to the lowermost part of the section, in deposits of the Reifling and Göstling formations (*Gladigondolella tethydis*; *Gladigondolella malayensis malayensis*, *Gladigondolella* sp.). The only exceptions in the Reingraben formation are ramiform conodonts accumulated in microcoprolites. The conodont association collected at the top of the Reifling Formation is represented by typical Julian forms, including *Paragondolella polygnathiformis, P. inclinata, P. foliata, P. tadpole, P. praelindae, Paragondolella* aff. *Par. foliata* (sensu^6^), *Gladigondolella tethydis, G. malayensis malayensis* and *“Mosherella” longnanensis* (Fig. 5). The presence of *P. polygnathiformis* constrains the age of the samples to Carnian, while *P. praelindae* together with *Paragondolella* aff. *Par. foliata* date the top of the Reifling Formation to the Julian 2^6,36^. Note that the genus *Gladigondolella* is a stenothermal species that prefers cool waters^12^, becoming extinct at the Julian/Tuvalian boundary^36^. The Color Alteration Index (CAI) of the conodonts equals 1, indicating a burial temperature below 50–80 °C^37^.

#### Palynology

**Figure 6:**
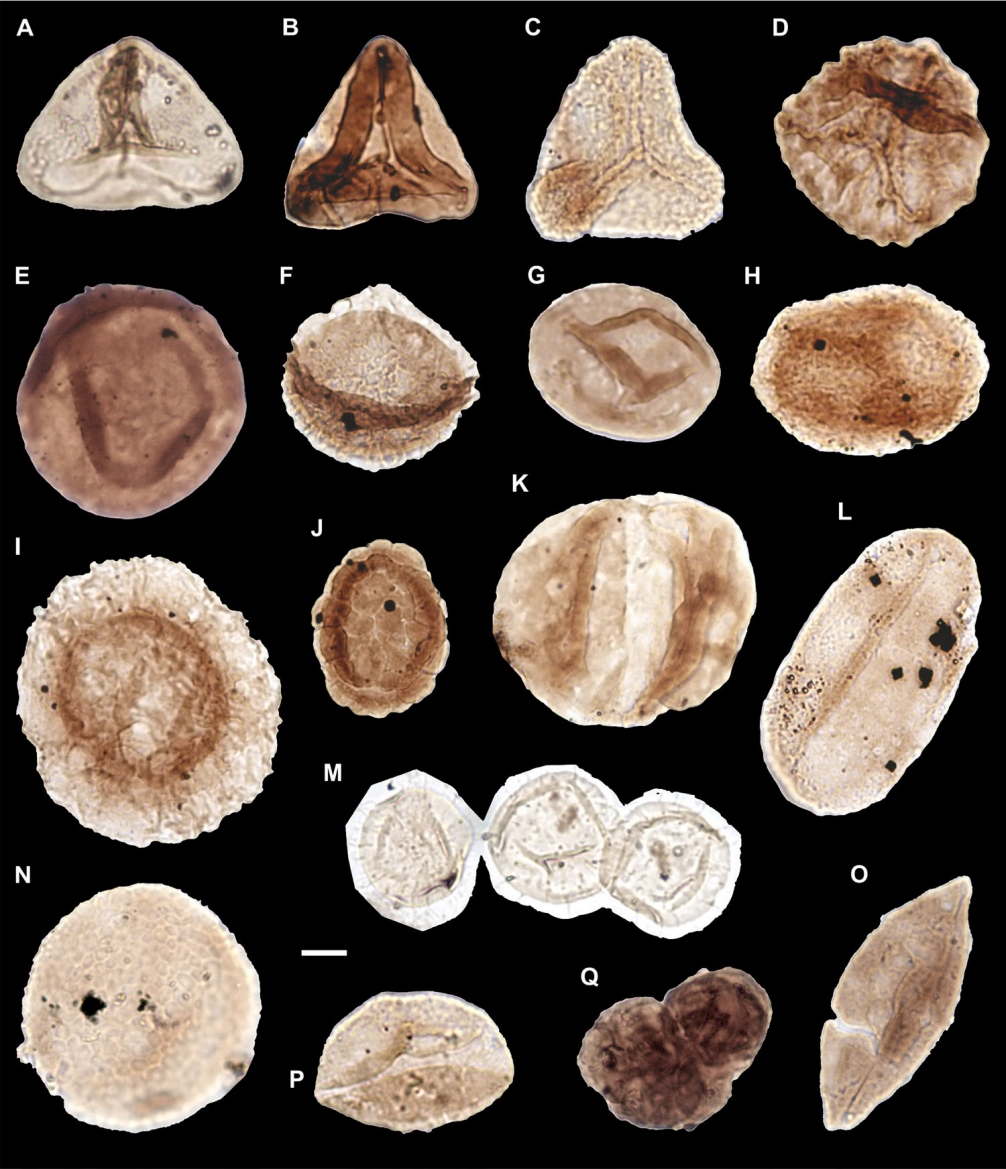
**(A**) *Concavisporites* sp., slide PO 0 T1; D38/2, NHMW 2021/0123/0527. (**B**) *Concavisporites* sp., slide PO340 T6; F31/3, NHMW 2021/0123/0528. (**C**) *Lunzisporites lunzensis*, slide PO 220, T4, G34/4, NHMW 2021/0123/0529. (**D**) *Lycopodiacidites* sp., slide PO 220, T4; J38/3, NHMW 2021/0123/0530. (**E**) *Laricoidites intragranulosus* PO 340, T5; E32/3, NHMW 2021/0123/0531. (**F**) *Kraeuselisporites* sp., slide PO 220, T4; J40/3, NHMW 2021/0123/0532. (**G**) *Aulisporites astigmosus*, slide PO60, T2; M46, NHMW 2021/0123/0533. (**H**) *Vallasporites ignacii*, slide PO 0, T1; G43, NHMW 2021/0123/0534. (**I**) *Patinasporites densus*, slide PO120, T3; H30, NHMW 2021/0123/0535. (**J**) *Camerosporites secatus*, slide PO 220, T4, M31/3, NHMW 2021/0123/0536. (**K**) *Alisporites* sp, PO120, T3, J46, NHMW 2021/0123/0537. (**L**) *Ovalipollis pseudoalatus*, slide PO 280, T5, G38, NHMW 2021/0123/0538. (**M**) *Micristridium* sp., slide PO 340 T6, M50, NHMW 2021/0123/0539. (**N**) *Tasmanites* sp., slide PO 0, T1, R31, NHMW 2021/0123/0540. (**O**) *Cycadopites* sp., slide PO 220, T4; X44, NHMW 2021/0123/0541. (**P**) *Cycadopites* sp., Slide PO 60, T2, F36, NHMW 2021/0123/0542. (**Q**) *Botryococcus*, slide PO 340 T6; G28, NHMW 2021/0123/0543. Scale bar 10 µm. Prepared by GR using CorelDraw X7; www.coreldraw.com.

The Reingraben formation contains terrestrial elements such as spores and pollen grains, and marine palynomorphs such as acritarchs, dinoflagellate cysts and chitinous remains including scolecodonts and foraminiferal linings (Fig. 6). Based on the palynological association the age is equal to the ammonoid *A. austriacum* Zone. The palynological composition corresponds to the *densus-maljawkinae* phase of Van der Eem^38^ and to the *Aulisporites astigmosus* assemblage^39^, corresponding to the *Duplicisporites continuus* assemblage of Roghi^40^.

No obvious changes in the palynological association were found within the Reingraben formation of Polzberg, while the *Lagenella martini* assemblage is present in the basal part of the overlaying as in introduction Lunz Formation^8,39^. The palynology here is characterized by the presence of a rich terrestrial association mainly represented by the typical Carnian sporomorphs of southern Europe^8,39,41–43^. Sporomorphs belonging to *Azonotriletes* (*Concavisporites, Calamospora, Todisporites, Punctatisporites, Lunzisporites, Verrucosisporites* and *Lycopodiacidites*), *Zonotriletes* and *Cavatomonoletes* (*Kraeuselisporites* and *Aratrisporites*, respectively) have been documented. The pollen association is composed of the monosaccites group characterized by the genera *Enzonalasporites, Vallasporites, Patinasporites* and *Pseudoenzonalasporites*, and of disaccites with the genera *Alisporites, Ellipsovelatisporites* and *Ovalipollis*. Among Circumpolles the main genera have been ascribed to *Camerosporites* and *Duplicisporites*. Monosulcates (*Cycadopites*) and Aletes are present but not abundant. Marine elements are composed by acritarchs (*Micrhystridium* sp.) and green algae belonging to the chlorophyta (*Tasmanites* spp.). The occurence of *Azonotriletes* and *Aulisporites* spp. reflects the presence of ferns, cycads and cycadalean swamp or marsh vegetation (floodplain vegetation), whereas the abundant presence of circumpolles and bisaccates indicate coastal pioneer vegetation and hinterland upland elments, respectively.

#### *Stable carbon isotopes-δ*^*13*^*C*_*carb*_

**Figure 7:**
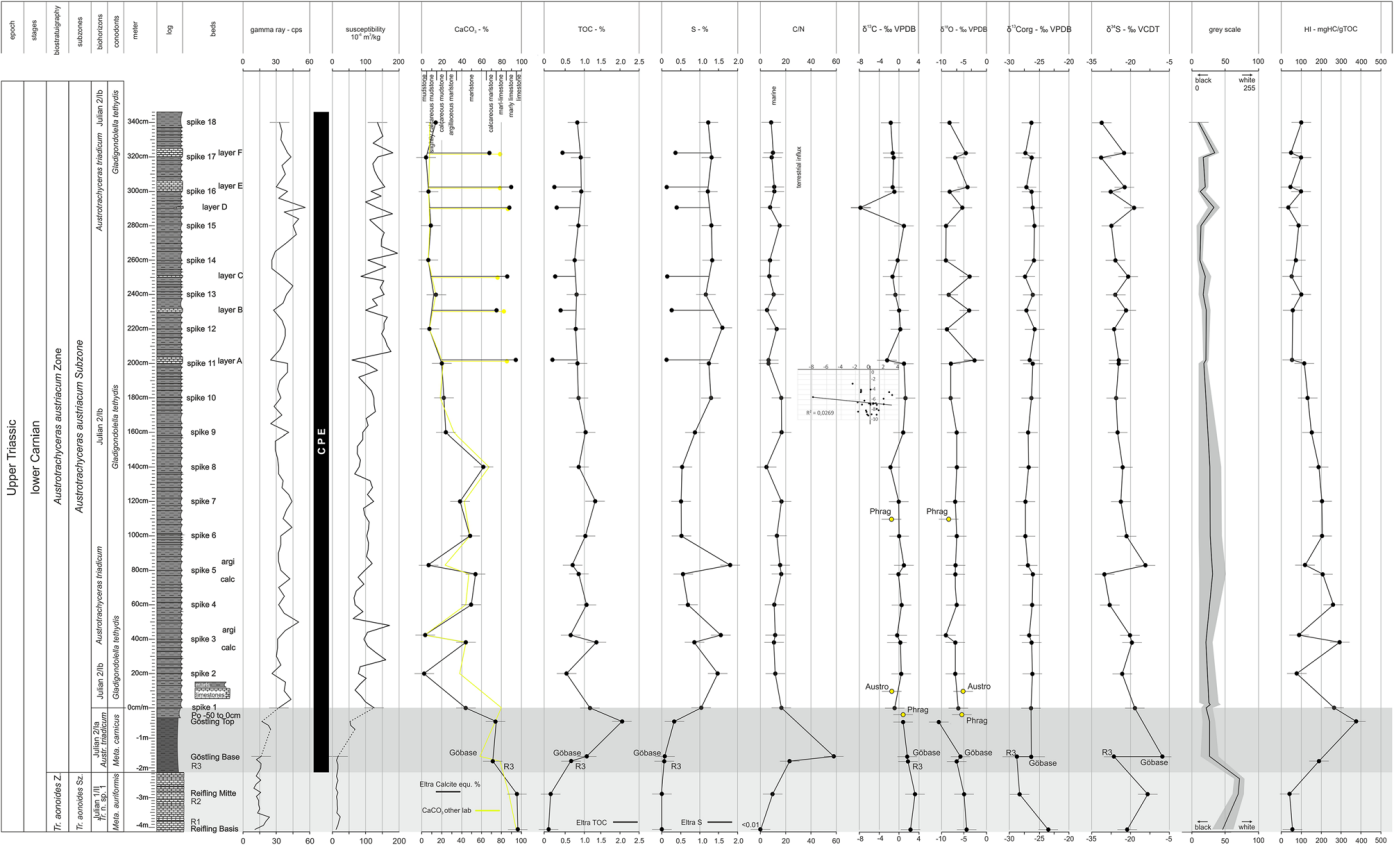
Compilation log (Po) and biostratigraphical, geochemical and geophysical data of the lower Carnian Polzberg locality. From left to right, ammonoid zonal scheme micro- and nannofossil data, logs and layer numbers, gamma-ray and magnetic susceptibility, CaCO_3_, total organic carbon (TOC), sulfur (S), carbon/nitrogen ratio (C/N); isotope bulk rock δ^13^C and δ^18^O, organic material δ^13^C, isotope bulk rock δ^34^S, grey-scale, and hydrogen index (HI). CPE, Carnian Pluvial Episode, range marked by a vertical black bar. Prepared by AL using CorelDraw X7; www.coreldraw.com.

The δ^13^C_carb_ values from the Polzberg section range from a minimum of − 7.89‰ (Po layer D in the Reingraben formation) to a maximum of + 3.05‰ (Po Reif R2; Reifling limestones) (Fig. 7, Supplementary Information), showing a stepwise significant negative trend. The δ^13^C_carb_ values in the Reifling limestones range between + 2.64 and + 3.05‰ with a mean value of + 2.84‰, which decrease in the Göstling formation to + 0.80 to + 1.89‰ (mean of + 1.52‰). Successively, the δ^13^C_carb_ values oscillate between − 1.72 and + 1.19‰ in the Reingraben formation, with a mean of − 0.04‰ (Fig. 7, Supplementary Table 1). The variation of δ^13^C_carb_ values is lower in the lower versus upper Reingraben formation. The average δ^13^C_carb_ value of intercalated dolostone layers is − 2.34‰, with one significatly more negative value of − 7.89‰ at Po layer D (shift of approx. 2–7‰).

δ^13^C_carb_ of primary aragonite from fossils (3 from Po section, 8 from historical collections) were also measured for comparison on ammonoid (*Austrotrachyceras* n 3, *Carnites* n 2, *Simonyceras* n 1), belemnoid (*Phragmoteuthis*, n 4) and gastropod (indet, n 1) shells. In situ found belemnoids have values ranging between − 1.59 and + 0.70‰, the in situ ceratitid ammonoid − 1.46‰. Regarding δ^13^C, fossils show no significant correlation with respect to the bulk rock (Fig. 7).

#### *Stable carbon isotopes-δ*^*13*^*C*_*org*_

Carbon isotope data of organic material (δ^13^C_org_) decrease significantly between the base of the Reifling limestones (− 23.4‰) and the lower part of the Göstling limestone (− 28.7‰) (Fig. 7, Supplementary Information). In the upper Göstling and lower Reingraben formations (< 80 cm), δ^13^C_org_ values are uniform (− 26.0 to − 26.7‰; average − 26.3‰), but slightly more negative between 80 and 200 cm (− 26.1 to − 27.3‰; average − 26.8‰). In the upper Reingraben formation (202–340 cm), carbonate-poor samples are characterized by less negative values (− 26.3 to − 25.7‰) than carbonate layers A to F (− 27.3 to − 27.2‰). Only the thin carbonate layer D has a value similar to that of the shales (− 26.1 ‰).

#### *Stable oxygen isotopes-*δ^18^O_carb_

Oxygen isotope (δ^18^O_carb_) bulk rock data from the Polzberg section (mean: − 6.75‰) range from − 10.55‰ (Po Goest Top) to − 2.82‰ (Po layer A in the upper Reingraben formation) (Fig. 7), showing no significant trend. The mean value in the Reifling limestones is − 4.79‰ (− 5.07‰ [Po Reif R2] to − 4.50‰ [Po Reif R1]), − 7.75‰ in the Göstling formation (− 10.55 to − 5.80‰), and − 6.78‰ in the Reingraben formation (− 9.14‰ [Po 260 cm] to − 2.28‰ [Po layer A]; Fig. 7, Supplementary Table 1). Relatively strong variations occur in the upper part of the section. The values of intercalated dolostone layers are significantly less negative (shift of 3–6‰), with a mean of − 4.19‰ (− 5.48‰ [Po layer D] to − 2.82‰ [Po layer A].

#### *Stable sulfur isotopes-δ*^*34*^*S*

δ^34^S values vary significantly in the Reifling limestones (− 20.89‰ [Po Reif R1] to − 13.34‰ [Po Reif R2] and decrease upwards from the base of the Göstling formation − 7.67‰ to the lower Reingraben formation (− 30.14‰ [Po 80 cm K]). Another upward decreasing trend occurs in the Reingraben formation between samples Po 80 cm T, clay [− 13.9‰] and Po 340 cm [− 31.87‰]; Fig. 7, Supplementary Table 1). The intercalated dolostone layers (mean − 21.69‰) show higher values (mean − 21.69‰; − 24.54‰ [layer A] to − 18.35‰ [layer D]) indicating a positive shift of ca. 5‰.

#### Grey-scale

Lower Carnian (Julian) grey-scale data (0 [black] to 255 [white]) of the Polzberg section steadily decrease from the grey Reifling limestone (mean: 73), to the dark laminated Göstling limestone (mean: 26.5), and the dark grey to almost black interval of the Reingraben formation (mean: 20.5; minimum: 11) (exclusive dolostones, layer A–F, Fig. 7). The values of the intercalated grey dolostone layers (A–F) range from 20 (layers C, D) to 34.5 (layer F).

#### Carbonate content

CaCO_3_ (calcite equivalent percentages) values of the Polzberg section (Po) range from 2.9% (Po 40 T, clay) to 94.7% (Po R1; Fig. 7, Supplementary Information), reflecting the presence of carbonate rocks and shales.

In the Reifling limestones at the base of the studied section, the values are very high (87–95%; Supplementary Table 1). In the Göstling limestones, they are lower (58–79%) and decrease towards the top. Values in the Reingraben formation (Fig. 7) decrease towards the more clayey top from ~ 80 to 6–7% in the uppermost layers. The intercalated grey dolostone layers contain 79.1% (layer F) to 86.9% (layer D) calcite_equivalent_. The decrease in the upper part of the section reflects the more clayey lithology. The correlation between the CaCO_3_ content and grey-scale is weak (correlation coefficient R^2^ = 0.39), with limestones being brighter than dark marls.

#### Total organic carbon and sulfur

Light grey limestones of the Reifling Formation contain very low amounts of TOC (0.09–0.15%) and sulfur (0.00–0.02%) (Fig. 7, Supplementary Information). Significantly higher TOC (0.67–1.97%) and sulfur (0.06–0.33%) contents are present in the laminated dark Göstling formation. The TOC values in the Reingraben formation (exclusive dolostone layers A–F) are on average 0.92%. They vary more strongly in their lower (0–140 cm; 0.59–1.32%) than upper part (160–340 cm; 0.76–1.04%). Maximum TOC occurs in carbonate-rich samples in the lower Reingraben formation (Po 40cmK, Po 80cmK), while TOC contents of dolostone layers A to F in the upper part are generally low (0.23–0.48%). Sulfur contents in Reingraben formation range from 0.51 to 1.78%, but are typically lower in dolostone layers (0.13–0.40%).

#### Rock–Eval data

These data are listed in the Supplementary Information. The HI value is plotted versus depth in Fig. 7. HI values of the Reifling limestones are very low (< 60 mgHC/gTOC), indicating the presence of kerogen Type III to IV. Values in the Gösting formation are higher, peaking at 403 mgHC/gTOC in the organic matter-rich sample “Göst. Top”. Based on HI, this sample contains kerogen Type II. HI values in the Reingraben formation vary between 35 and 293 mgHC/gTOC. They vary strongly in the lower 80 cm of the Reingraben formation. Here, samples with carbonate contents < 10% are characterized by low HI (78–120 mgHC/gTOC), while samples with higher carbonate contents (38–54%) have significantly higher values (200–300 mgHC/gTOC; Type III-II kerogen). These values decrease gradually from 206 to 116 mgHC/gTOC (Type III kerogen) in the interval between 100 and 200 cm. Above this level, HI is generally low, but even lower in dolostone layers (35–57 mgHC/gTOC) than in low-carbonate layers (70–101 mgHC/gTOC). The average T_max_ and PI of samples with S2 values > 0.5 mgHC/g rock is 431 °C and 0.03, respectively.

#### Total nitrogen

TN was measured to determine the C/N ratio (calculated as TOC/TN) (Fig. 7; Supplementary Table 1). C/N values (weight-based) in the Polzberg section are generally low (0–9.7) in the limestones of the Reifling Formation, with a strong increase up to 23.2 and 58.2 in the Göstling formation, and again lower, oscillating values around a mean of 11.1 with maximum 16.7 and a minimum of 4.9 in the Reingraben formation. C/N values in the dolostone layers are generally low (mean 7.9; maximum 10.9 in layer Po layer E, minimum 5.2 in Po layer B).

#### Molecular composition of hydrocarbons

**Figure 8:**
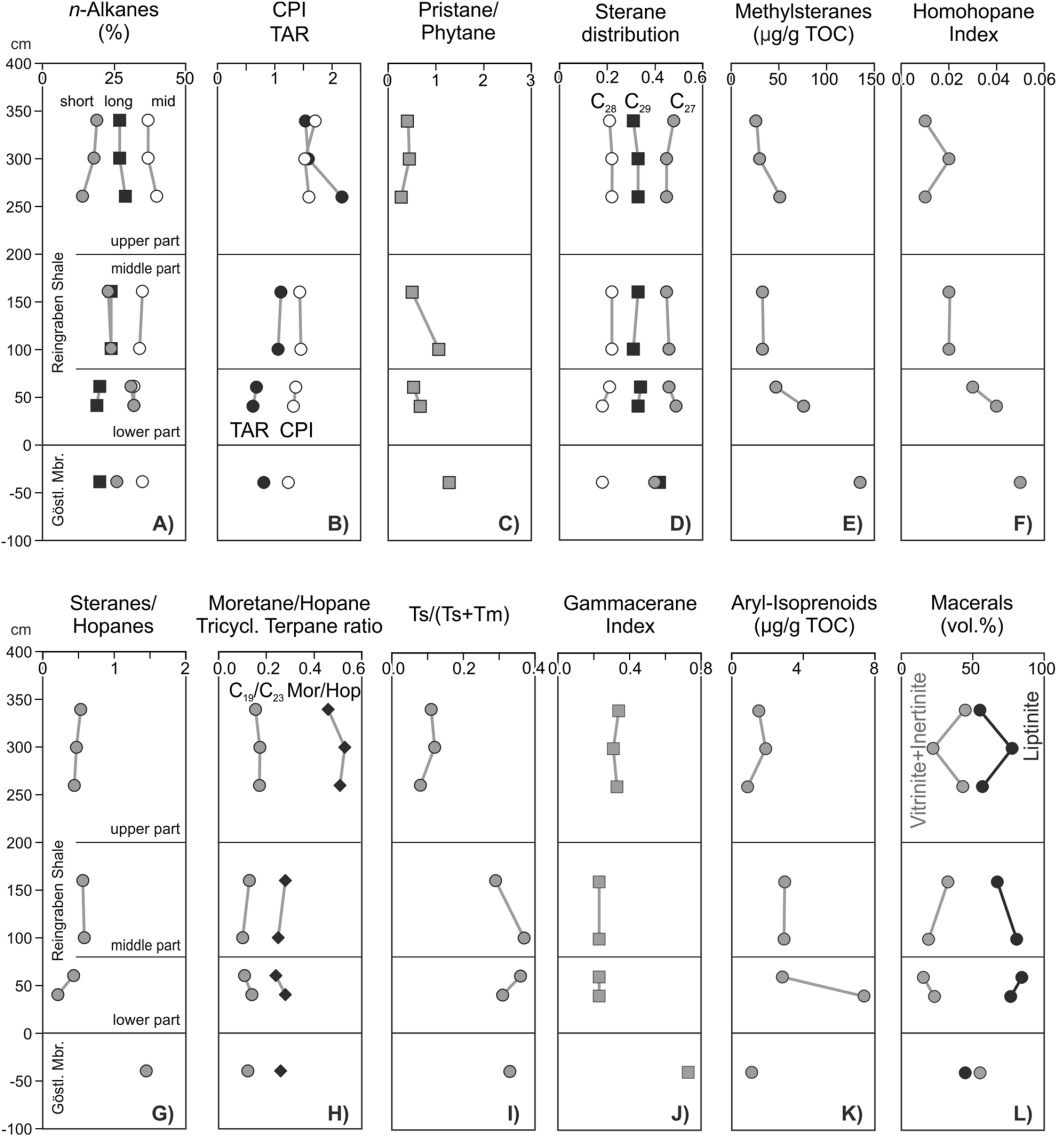
Concentrations and ratios of selected organic compounds (A-K) and maceral percentages (L). Short-chain *n*-alkanes: 100*n-C_15–19_/Σ *n*-alkanes; mid-chain *n*-alkanes: 100*n-C_21–25_/Σ *n*-alkanes; long-chain* n*-alkanes: 100*n-C_27–31_/Σ *n*-alkanes; CPI: Carbon Preference Index^45^, TAR: terrestrial/aquatic ratio (*n*–C_27+29+31_/*n*–C_15+17+19_^44^); sterane distribution: C_27_/C_27–29_ steranes, C_28_/C_27–29_ steranes, C_29_/C_27–29_ steranes. Prepared by RS using CorelDraw X7; www.coreldraw.com.

Rock extracts from the organic matter-rich sample from the Göstling formation (Göst Top; 1.97% TOC) and seven samples from the Reingraben formation with relatively high organic matter contents (0.76–1.32% TOC) were investigated regarding their biomarker distribution. Concentrations and ratios of selected organic compounds are plotted versus stratigraphic height in Fig. 8 and are listed in the Supplementary Information.

##### *n*-Alkanes and isoprenoids

*n*-Alkanes are abundant in all samples (0.3–2.4 mg/gTOC). Mid-chain forms (100*n-C_21–25_/Σ *n*-alkanes) are dominant in all samples (32–40%), but significant differences exist in the percentage of short-chain (100*n-C_15–19_/Σ *n*-alkanes) and long-chain forms (100*n-C_27–31_/Σ *n*-alkanes; Fig. 8A).

- In samples from the Göstling formation and the lower Reingraben formation (40–60 cm), the amount of short-chain *n*-alkanes is higher (26–32%) than that of long-chain *n*-alkanes (*n*-C_27–31_: 19–20%);
- In samples from the middle part of the Reingraben formation (100–160 cm), short- and long-chain n-alkanes occur in similar amounts (23–24%);
- In samples from the upper part of the Reingraben formation (260–340 cm), long-chain *n*-alkanes are significantly more abundant (27–29%) than short-chain *n*-alkanes (14–19%).

The upward increase in the relative proportion of long-chain *n*-alkanes is also evident in the ratio *n*–C_27+29+31_/*n*–C_15+17+19_, the so-called terrestrial/aquatic ratio (TAR^44^). This ratio is significantly below 1 in the Göstling and lower part of the Reingraben formation; about 1 in the middle part of the Reingraben formation; and significantly above 1 in the upper part (Fig. 8B).

The carbon preference index (CPI; according to Bray and Evans^45^) increases gradually upwards from 1.24 to 1.71 (Fig. 8B). Positive correlations exist between CPI and the amount of long-chain *n*-alkanes (R^2^ = 0.79) and between CPI and TAR (R^2^ = 0.65).

The pristane/phytane (Pr/Ph) ratio^46^ is slightly above 1 in the sample from the Göstling formation (1.28) and in the Reingraben sample at 100 cm (1.06), and is significantly lower (0.27–0.67) in the remaining samples (Fig. 8C).

##### Steroids

Sterane concentrations range from 10 to 70 mg/g EOM (extractable organic matter). Concentrations of C_27_ and C_29_ steranes are similar (~ 40%) in Göst Top, but C_27_ steranes are significantly more abundant (45–49%) than C_29_ steranes (31–34%) in all Reingraben samples. C_28_ steranes are generally less abundant (18–22%; Fig. 8D). This is also reflected by low C_28_/C_29_ sterane ratios (0.43–0.71).

The C_29_ sterane 20S/(20S + 20R) ratio and C_29_ sterane αββ/(αββ + ααα) ratio are maturity parameters^47^ and range from 0.23 to 0.35 (avg. 0.29) and from 0.23 to 0.28 (avg. 0.26), respectively (Supplementary Information).

C_27_ Diasteranes are present in all samples in lower amounts than their C_27_ regular sterane counterparts. The C_27_ diasterane/C_27_ regular sterane ratio varies between 0.29 and 0.42 and is especially high in the Göst Top samples.

4-Methylsteranes were detected in all samples. Their concentration peaks (135 µg/gTOC) in the Göstling formation sample and decreases upwards from 76 to 26 µg/gTOC within the Reingraben formation (Fig. 8E; Supplementary Information).

##### Hopanoids and related compounds

The dominant non-aromatic cyclic triterpenoids are hopanes (23–186 mg/g EOM) (Supplementary Information). The sterane/hopane ratio is high in the Göstling sample (1.45) and significantly lower (0.21–0.58) in the Reingraben formation, where it shows a subtle upward increase (Fig. 8G). 22S/(22S + 22R) isomer ratios of αβ C_31_ hopane range from 0.46 to 0.57 (Supplementary Information). The moretane/hopane ratio ranges from 0.24 to 0.53 and is relatively high (> 0.45) in the upper part of the studied section (Fig. 8j).

The C_35_ homohopane index (HHI = C_35_/(C_31_ − C_35_) homohopanes^48^) decreases upwards from 0.05 to 0.01 (Supplementary Table 1; Fig. 8F). The Ts/(Ts + Tm) ratio varies between 0.08 and 0.37 and is very low (≤ 0.12) in the upper part of the studied section (Fig. 8I). The gammacerane index (10*gammacerane/gammacerane + C_30_ hopane^49^) is high in Göst Top (0.73), low in the lower part of the Reingraben formation (0.23) and slightly higher in its upper part (0.31–0.34) (Fig. 8J).

Tricyclic terpanes (TT) occur at relatively high concentrations (3–19 μg/g EOM). The dominant TT is C_23_ TT as reflected by low C_19_/C_23_ TT (0.10–0.17) and C_24_/C_23_ TT ratios (0.30–0.71) (Supplementary Information). Relatively high C_19_/C_23_ TT (0.10–0.17) occur in the upper part of the Reingraben formation (Fig. 8H).

##### Aromatic hydrocarbons

The samples contain benzenes, napthalenes, phenanthrenes and their alkylated equivalents in the aromatic fraction. Dibenzothiophenes are largely missing. Alkylphenanthrenes are present and include methylphenanthrene (MP) and dimethylphenanthrene (DMP). The methylphenanthrene index (MPI-1^50,51^) ranges from 0.50 to 0.68.

C_13_ to C_22_ aryl isoprenoids are present in all samples (0.9–7.4 µg/gTOC; Fig. 8K). The concentration is low in Göst Top, high near the base of the Reingraben formation and decreases upwards within the latter unit. The ratio between C_13-17_ and C_18-22_ aryl isoprenoids (aryl isoprenoid ratios (AIR^52^) is typically low (< 0.21). In contrast to aryl isoprenoids, isorenieratane was not detected.

#### Magnetic susceptibility and gamma-ray profiles

The magnetic susceptibility (χ) and gamma-ray (GR) values differ markedly between the lower calcareous part of the Reifling and Göstling limestones (*Trachyceras aonoides* Zone) and the marly to shaly upper part (*Austrotrachyceras austriacum* Zone) of the Polzberg section, showing a significant trend throughout the entire section (Fig. 7, Supplementary Information). Low gamma-ray values (in cps) of the Reifling limestone log oscillate around a mean of 14.8, with a maximum of 23 at Po/R1 at − 3.7 m and minimum of 10 at Po/R2 at − 2.7 m (Fig. 7). Slightly increased gamma-ray values of the Göstling limestone log show a mean of 16.7, with a maximum of 25 at Po/R1 at − 3.7 m and minimum of 12 at Po/R2 at − 2.7 m (Fig. 7). Remarkably higher gamma-ray values of the Reingraben formation oscillate around a mean of 35.8 (maximum 56 at Po/layer D, minimum 25 at Po/170 cm and 210 cm), without a significant trend (Fig. 7).

Low magnetic susceptibility values (SI) of the Reifling limestone log oscillate around a mean of 4.75 × 10^−6^ m^3^/kg, with a maximum of 13 × 10^−6^ m^3^/kg at Po/R1 at -3.7 m and minimum of 2 × 10^−6^ m^3^/kg at Po/R2 at -3.5 m and -2.7 m (Fig. 7). The susceptibility values are remarkably higher in the Göstling limestone log (27.5 × 10^−6^ m^3^/kg, maximum 68 × 10^−6^ m^3^/kg at Po/Göst Top, minimum 7 × 10^−6^ m^3^/kg at Po/Göst base R3) (Fig. 7). Strongly increased susceptibility values of the Reingraben formation oscillate around a mean of 116.7 × 10^−6^ m^3^/kg, with a maximum of 195 × 10^−6^ m^3^/kg at Po/270 cm and minimum of 59 × 10^−6^ m^3^/kg at Po/layer A, with a significant positive trend toward the more shaly top (Fig. 7). Lowermost values are lower in the calcareous dolostone (layers A-F), fluctuating between 59 × 10^−6^ m^3^/kg in layer A at Po/210 cm and 181 × 10^−6^ m^3^/kg in layer F at Po/310 cm. In the limestone interval of the Reifling and Göstling formations, gamma ray values are weakly correlated with susceptibility (R^2^ = 0.37). In the marly and shaly Reingraben formation, gamma ray values are not correlated with susceptibility (R^2^ = 0.031).

#### Maceral analysis

Because of the generally low organic matter content, the maceral analysis can be at best considered semi-quantitative. Nevertheless, percentages of maceral groups are presented in Fig. 8l. This figure shows that liptinite is the prevailing maceral group. Liptodetrinite and alginite are the main liptinite macerals, but sporinite occurs as well. Vitrinite and inertinite (including recycled vitrinite) are present in subordinate amounts. Overall, the organic matter is of autochthonous marine and allochthonous terrigenous origin.

### Interpretation

#### Thermal maturity

Rock–Eval parameters T_max_ (average 431 °C) and PI (0.03) show that the organic matter is thermally immature. Maturity-related biomarker parameters (20S/(20S + 20R) ratio of C_29_ steranes: avg. 0.29; 22S/(22S + 22R) ratio of C_31_ hopanes: avg. 0.52) suggest a maturity at the transition from immature to early oil window. This agrees with vitrinite reflectance values of nearby coals from the overlaying as in introduction Lunz Formation (0.45–0.71%Rr^53^) and of a nearby drift wood (Sau1 “Gagat”; 0.72 ± 0.03%Rr). In any case, a significant influence of diagenesis on organic and inorganic parameters can be excluded.

#### Organic matter richness

The light grey limestones of the Reifling Formation contain very low amounts of TOC (≤ 0.15%), suggesting poor conditions for organic matter preservation. Hence this interval is not further discussed here.

The laminated dark Göstling formation contains partly high TOC contents (max. 1.97%). TOC/S ratios of these samples are typically high (> 2.8), suggesting sulfur limitation. TOC and carbonate content are not correlated. With relation to organic matter characteristics, three intervals can be distinguished within the Reingraben Shale.

**Figure 9:**
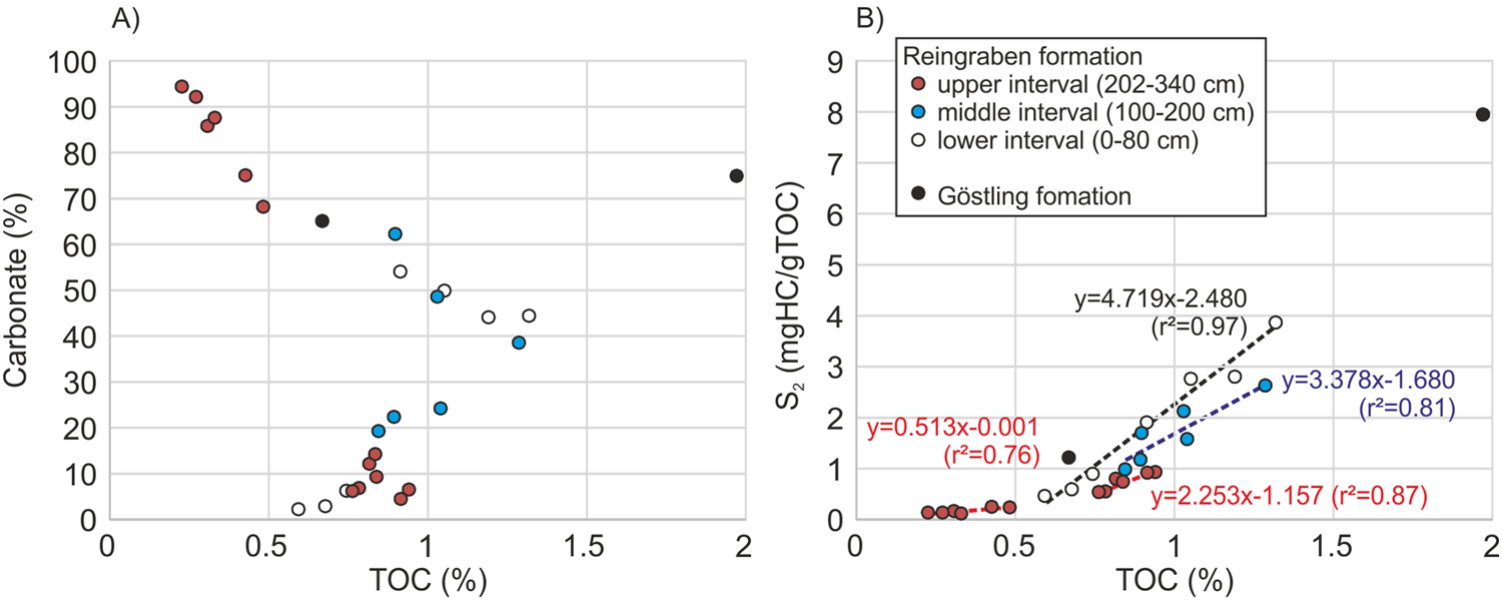
Cross-plots of (**A**) calcite equivalent percentages and (**B**) Rock–Eval parameter S2 versus TOC. The slope of the regression lines indicates the average HI of different organic matter populations. Prepared by RS using CorelDraw X7; www.coreldraw.com.
  S2 values show a strong correlation with TOC (R^2^ = 0.97; Fig. 9B). The slope of the regression line suggests that the true HI is in the order of 470 mgHC/gTOC and significantly higher than those calculated from measured S2 values. This points to a strong mineral matrix effect^54^ or the presence of two populations of organic matter: inert and reactive OM^55^. As data from different lithologies plot along the regression line, the data likely reflect the presence of largely inert, probably terrigenous OM (~ 0.5 %) and varying amounts of aquatic OM with a HI of ~ 470 mgHC/gTOC.
  The general positive relation between TOC and carbonate contents for low carbonate samples (Fig. 9A) suggests that the amount of aquatic OM may be controlled by the productivity of calcareous organisms. In contrast, the high percentage of carbonate minerals in marly rocks results in dilution of OM (and a negative relation between calcite and TOC; Fig. 9A).
- The middle interval (100–200 cm) is characterized by a gradual upward decrease in TOC (1.29–0.85) and HI (206–116 mgHC/gTOC). The general TOC-calcite and TOC-S2 relations are similar to those in the lower interval (Fig. 9A). They show that the change of a positive effect of carbonate content on TOC to a negative diluton effect is at about 30% carbonate. The average HI of the aquatic organic matter in the middle interval is somewhat lower (~ 340 mgHC/gTOC) in the middle than in the lower interval (Fig. 9B).
- The upper interval (202–340 cm) is characterized by the presence of thin carbonate layers with very low TOC contents (< 0.5%) intercalated into low carbonate background sediments with low to moderate TOC (0.76–0.94%). In this interval the dilution of OM by carbonate minerals is especially evident (Fig. 9A). The HI of the aquatic OM is ~ 225 mgHC/gTOC, while the HI of the “inert” OM may be on the order of 50 mgHC/gTOC (Fig. 9B), suggesting the dominance of partly inert terrigenous OM in carbonate-rich layers.

- The lower interval (0–80 cm) is characterized by strongly varying but locally relatively high TOC (0.59–1.32%) and HI values (78–293 mgHC/gTOC; Fig. 7). Clear differences exist between the abundance of OM in mm-thin shaly (< 10% carbonate) and marly layers (44–55% carbonate). TOC contents are low in shaly layers (< 0.8%), but increase with carbonate contents (Fig. 9A). In contrast, TOC contents are higher in marly layers (0.9-1.4%), but decrease with increasing carbonate content.

#### Origin of organic matter

We used molecular parameters, Rock–Eval parameter HI, and maceral data to determine the organic matter type in samples from the uppermost Göstling and the Reingraben formations.

Epicuticular waxes produce long-chain *n*-alkanes with a significant odd-to-even preference. Thus, both the ratio of long- to short-chain *n*-alkanes (TAR^44^) and CPI^45^ are often used to determine the relative amount of terrigenous organic matter^56^. This approach is also supported by the observed positive correlations between CPI and TAR (R^2^ = 0.65) and between CPI and the amount of long-chain *n*-alkanes (R^2^ = 0.79). Since CPI and TAR reflect the relative contribution of land-plants, it is unsurprising that HI is negatively correlated with TAR (R^2^ = 0.52) and CPI (R^2^ = 0.71).

Based on these observations, significant variations in organic matter input occurred during deposition of the studied interval:

- The organic matter in the uppermost Göstling and lower Reingraben formation (< 80 cm) is characterized by a high amount of aquatic organisms mixed with land-plant derived organic matter. While lamalginite is present, petrographic evidence for extensive algal or bacterial mats is missing. Interestingly, the Göstling sample shows the highest concentration of C_29_ steranes, often attributed to land-plants. However, C_29_ steranes may also be produced by other organisms^56^. Furthermore, the content of liptinite macerals in the Göstling sample is surprisingly low. Considering that the total amount of macerals identified in this TOC-rich sample is lower than expected, much of the organic matter may not be visible under the microscope. While TAR and CPI are similar in the Göstling and the lower Reingraben samples (Fig. 8B), the steranes/hopanes ratio is much higher in Göstling (1.45 versus 0.21; Fig. 8G). Because hopanes are produced by prokaryotic organisms, this suggests a significantly higher contribution of bacterial biomass in the lower Reingraben formation.
- In the middle part of the Reingraben formation (100–200 cm), HI is reduced (Figs. 7, 9), suggesting a decrease in aquatic organic matter. This interpretation is supported by the observed moderately high TAR and CPI values in this interval. Slightly increased steranes/hopanes ratios (0.56–0.58; Fig. 8G) suggest a decrease in bacterial activity compared to the lower part of the Reingraben shale.
- The upper interval at Reingraben (202–340 cm) displays the lowest HI (Figs. 7, 9) and the highest TAR and CPI values (Fig. 8B). This is clear evidence for a dominance of land-plant derived organic matter. This interpretation also agrees with the elevated C_19_/C_23_ tricyclic terpanes ratio^57^ (Fig. 8H) and a relatively high abundance of terrigenous vitrinite and inertinite macerals (Fig. 8L).

Overall, the relative proportion of terrigenous OM increases from the Göstling formation to the lower, middle and upper intervals of the Reingraben formation. Since the TOC contents decrease in the same direction, this reflects either a gradual decrease in marine OM productivity or a dilution of marine OM due to increased clastic input rather than an absolute increase in terrigenious OM. High concentrations of 4-methylsteranes in Göstling and the lower Reingraben formation (Fig. 8e) suggest that dinoflagellates contributed to the aquatic biomass^58,59^ (see also palynological results).

#### Redox and salinity conditions

The Pr/Ph ratio^46^ and the homohopane index (HHI^48^) are widely used redox parameters. Moreover, the concentration of aryl-isoprenoids has been used to determine photic zone anoxia^52^. The determined Pr/Ph ratios decrease from the Göstling to the upper Reingraben formation from 1.3 to 0.4 (Fig. 8c). This suggests dysoxic conditions in the lower part of the studied section and strictly anoxic conditions in the upper part. In contrast, the HHI suggests a gradual upward decrease in the degree of anoxia (Fig. 8F), which is also indicated by the stratigraphic trend of aryl-isoprenoids within the Reingraben formation (Fig. 8K). However, aryl-isoprenoids are rare in the sample from the top of the Göstling formation. Summarizing, different redox parameters show different depth trends. Although this may reflect temporally different redox conditions in different portions of the water column, these results should not be over-interpreted. Nonetheless, the laminated sediments were clearly deposited under oxygen-depleted conditions^20,21,24^.

The oxygen depletion was probably related to salinity stratification^20,21,24^. This interpretation is supported by high Gammacerane Index (GI) values, which peak (0.73) in Göstling formation but are also high in Reingraben samples (0.23–0.34; Fig. 8J). Interestingly, GI is slightly higher in the upper than in the lower and middle intervals of the Reingraben formation.

The moretane/hopane and the Ts/(Ts + Tm) ratios show a very strong negative correlation (R^2^ = 0.96). Both ratios are maturity parameters, but are also strongly facies dependent^56^. For example, alkaline conditions favour formation of moretane over hopane and Tm over Ts^56^. Hence, high moretane/hopane and low Ts/(Ts + Tm) ratios in the upper Reingraben formation (Fig. 8H, I) may reflect an increase in pH. However, as both ratios are also highly correlated with C_19_/C_23_ tricyclic terpanes ratios (Fig. 8H; R^2^ = 0.89 and 0.88, respectively), an influence of organic matter type cannot be excluded.

#### Bacterial sulfate reduction

Very low δ^34^S values are characteristic for biogenic sulfate reduction (BSR). The vertical distribution of δ^34^S values suggests two upward trends with increasing degree of BSR for the Reingraben Shale (Fig. 7). A slightly lower degree of BSR is indicated for the dolomitic layers. The degree of BSR can be estimated independently using the Sulfate Reduction Index (SRI = (TOC + TS/1.33)/TOC) of Lallier-Verges et al.^60^. This value is high for Reingraben Shale samples (1.30–2.85).

#### Evolution of the Polzberg succession

The depositional environment was influenced by the evolution of climate and environmental conditions. The sequence shows three main lithologies and two main lithological transitions with the grey radiolaria limestones of the Reifling Formation, the transitional laminated black silicious limestones Göstling formation and the laminated argillaceous shaly Reingraben formation. Similar lithological sequences have been observed in Upper Triassic successions of several other localities in the NCA of Austria (e.g., Göstling, Grossau, Mendlingbach, Saugraben, Scheiblinggraben, Steinbachgraben, Rehberg^10,22^). Although minor differences occur in the lithotypes and in their thicknesses, the lithological boundaries occur consistently at a regional scale. This is an evidence for a change in basinal sedimentation throughout the NCA and the western Tethys during the Late Triassic.

The grey-scale factor is indicative of a higher detrital content, marking an increased siliciclastic input in the clay versus the lower limestone deposits, especially in the Reingraben formation^4^. Grey scale values show a positive correlation with CaCO_3_ contents (i.e. light grey with high carbonate, dark gray to black with low carbonate; R^2^ = 0.39) and negatively correlate with TOC values as bright colours in the Reifling Formation and intercalated layers A-F correspond to low TOC (R^2^ = 0.14) and S values (R^2^ = 0.33). Dark lithologies go with increased TOC and S values respectively. The CaCO_3_ content from rock samples (Po Reif base to Po 340 cm, Fig. 7) reflects a change from a radiolarian limestone-dominated (Reifling and Göstling formations) to a more argillaceous marly facies (Reingraben formation). These results corroborate those obtained from gamma-ray (GR) and magnetic susceptibility logs from the calcareous basal Reifling Formation over the dark laminated limestone of the Göstling formation to the arigillaceous mudstones of the Reingraben formation. Increasing values in GR and MS hint at a higher clay mineral content, supported by thin sections and microfacies analysis suggest an environment with fluctuating carbonate productivity or oscillating siliciclastic and terrestrial input.

Note that the correlation between TOC and N is very poor (e.g. R^2^ = 0.28 for Reingraben formation), potentially indicating that some N is non-organic. Organic nitrogen derives mostly from peptides and amino acids of planktonic organisms^61^ and can therefore be used as an indicator for planktonic productivity. These data are comparable to modern data by ^62–66^ who showed that the distribution and abundance of N are correlated to TOC. Nitrogen availability often limits biological production, both in terrestrial and in marine environments. Total nitrogen (TN) was determined in order to calculate C/N (TOC vs. TN) ratios. The C/N ratio reflects the origin of organic matter: organic matter (OM) of terrestrial origin typically results in C/N > 15, while marine OM features ratios of ≤ 5^65,67,68^. The low C/N ratio in the lowermost part of the Polzberg section within the light grey calcareous Reifling Formation suggests dominant marine organic matter and an increasing terrestrial influx in the upper part of the dark-laminated Göstling and argillaceous Reingraben formation, in agreement with the deposition on a continental margin.

Palynomorphs from the top Reifling Formation (Julian 1), over the Göstling (Julian 2/Ia) and Reingraben formations (Julian 2/Ib) to the siliciclastic Lunz Formation (Julian 2/II), show a trend from dryer climate to warmer, more humid conditions^8,39^. A temperature maximum was detected for the Reingraben formation after a positive shift of 2 °C at the transition from the Göstling to Reingraben deposits^8^. The Reingraben formation is characterized by terrestrial and marine palynomorphs (spores, pollen and acritarchs versus dinoflagellate cysts, respectively). Spores belong to ferns, cycads and cycadaleans and are indicative of swamp or marsh vegetation. The abundant presence of gymnosperm pollen grains (circumpolles and bisaccates) indicates coastal pioneer vegetation and hinterland upland elements. Acritarchs and chlorophyta are the marine elements. The palynomorphs show a transition from xerophytic to more hygrophytic assemblages, reflecting a climate change during the onset of the CPE.

The δ^13^C_bulk_ values (Fig. 7) are coherent with the evolutionary stable isotopic trends of normal marine deposits measured in other, comparable Carnian sections within the Tethyan Realm. In particular, the δ^13^C_carb_ values in the Reifling limestones have a mean of + 2.84‰, decrease in the Göstling formation to a mean of + 1.52‰, and oscillate in the Reingraben formation (excl layers A–F) around a mean of − 0.04‰ (Fig. 7, Supplementary Information). This negative shift of the mean value of ca. 1.5‰ δ^13^C_bulk_ values in the lower Carnian Polzberg section occurs in the transitional zone between the *Trachyceras aonoides* and *Austrotrachyceras* zones (i.e. Julian1 to Julian 2^8^).

The δ^13^C_org_ values from the Lunz am See area including the Polzberg section were described in a study on carbon isotope records to examine carbon cycle perturbation from the Southern Alps (Dolomites, Italy) and the Transdanubian Range (Hungary) (Dal Corso et al.)^7^. This transitional phase, the Julian 1-Julian 2 boundary interval^7^ mirrors the onset of the Carnian Pluvial Episode, manifested in the first negative carbon isotope excursion (CIE 1). This shift can reach 4‰ in terrestrial and biomarker material, and approx. 2‰ in bulk organic matter.

δ^13^C_org_ values in the Reifling limestones exhibit a mean of − 25.85‰, in the Göstling formation with a mean of − 27.52‰, and oscillate in the Reingraben formation around a mean of − 26.49‰ (Fig. 7, Supplementary Information). Hence, a negative shift of the mean of ca. 1.67‰ δ^13^C_org_ in the lower Carnian Polzberg section reflects the transitional zone of Julian1 to Julian 2, followed by a slight positive shift of ca. 1.03‰. The shifts can be compared and are in the range of previously described fluctuations^2,7,8,69^. A negative shift of about 3.8‰ δ^13^C_org_ was reported for the transition from the topmost Reifling Formation to the Göstling formation. δ^13^C_org_ from the Polzberg section ranges from − 28.75 to − 23.45‰ (max range 5.3‰), similar to ranges given by Dal Corso et al.^7^ with − 28.4‰ to − 21.8‰ (max range 6.6‰; compiled section Göstling and Polzberg) and by Mueller et al.^8^ with − 28.5‰ to − 23.1‰ (max range 5.4‰; compiled section Göstling; Großreifling and Polzberg). These perturbations in carbon curves and dataset are linked to major changes in marine sedimentology, hence lithology, caused by an abrupt rise in rainfall and terrestrial weathering at the onset of the CPE. Intensified Pangaean monsoon activity led to enhanced terrestrial and freshwater influx into basinal environments, caused by the Wrangellia vulcanism, a Large Ingneous Province with mega flood basalts^1,2,7,8,14^. The negative shift in NCIE-1 (= CIE 1) mirrors an enhanced injection of δ^13^C-depleted CO_2_ into the atmosphere-oceanic system^8–10,70,71^. At Polzberg, the onset of the Göstling formation with calciturbiditic, radiolaria limestones is contemporaneous with the demise of the Wetterstein carbonate platform, coinciding with a negative shift in δ^13^C_carb_ of around 2‰ around this transition from the basal peloidal-filamentous Reifling Formation, over the laminated radiolaria-rich Göstling formation to the lowermost 5 m of the argillaceous Reingraben formation^8,10^. Note that a coeval rise of CCD was documented in the Lagonegro Basin (southern Italy) ^6^.

The carbon isotope values are consistent with those observed in other Upper Triasic records of the western Tethyan Realm (Austria, Germany, Hungary, Italy, Spain, Tunisia, Turkey). The herein presented δ^13^C record exhibits the typical range of values for Carnian hemipelagic to basinal deposits, which in the present case are basal normal marine carbonates to brackish-influenced shales at the top. Single peaks and trends in the δ^13^C record are also important for correlation with other reference sections.

The very low δ^18^O_carb_ values ranging from − 5.07 to − 4.50‰ (Reifling Formation), − 10.55 to − 5.80‰ (Göstling formation) and − 9.14‰ to − 2.28‰ (Reingraben formation) at Polzberg are the result of diagenetic overprint. The values are thus not representative for paleotemperature calculation or stratigraphical correlation.

The variation of χ and GR within the sedimentary record of the Polzberg section is a direct function of the variation of the mineral contents (e.g., clay minerals, magnetic minerals). χ includes contributions from all minerals (diamagnetic, paramagnetic and ferrimagnetic) present in the sediment in proportion to their abundance. χ and GR values reflect the direct function of the terrigenous and siliciclastic input into the marine Reifling Basin. The considerable rise in χ and GR values from the basal Reifling limestones, over the Göstling limestone to the argillaceous and shaly lithologies of the Reingraben formation mirror the change of climatic conditions during the Julian 1/Julian 2 interval, with increased temperatures, humidification and terrestrial and freshwater influx. Subsequently, higher radioactivity values reflect a higher clay content toward the top of the section. Climatic conditions during the CPE with enhanced rainfall triggered erosive mechanisms on already existing terrestrial areas. This boosted the terrigenous, brackish and freshwater influx into intraplatform basins. These processes were associated with an enrichment of radiant minerals and trace elements. The peak if this redepositional phase was marked by the climax of siliciclastic influx at the Julian 2/II with the prograding deltaic deposition and total infill of the Reifling Basin with sandstones of the Lunz Formation (with famous plant fossils and coals).

## Conclusions

Integrated high-resolution studies on the Polzberg section reveal the early Late Triassic paleoceanographic history of the intraplatform Reifling Basin area. Paleontological, lithological, geochemical and geophysical data show evidence for deposition during the onset of a humid and warmer episode in the Carnian, known as the Carnian Pluvial Episode (CPE). A comprehensive analysis of the Polzberg sections from the Northern Calcareous Alps established a reference section in the western Tethyan region, including the Polzberg *Konservat-Lagerstätte*. The documented lithological variations reflect the paleoenvironmental and climate change during the lower Carnian *Austrotrachyceras austriacum* Zone (Julian 2; Car 2). A lithological change from carbonate-dominated limestones, over black laminated limestones up to arillaceous to shaly deposits appears to be triggered by the Wrangellian Large Igneous Province (LIP). The enhanced LIP volcanism increased emissions of volcanic gases into the atmosphere, resulting in positive shifts of temperatures and rainfall along with negative shifts of δ^13^C. Carbonate systems (i.e. sedimentation) were interrupted or disappeared, terrestrial and freshwater influx increased, subsequently forming anoxia in basinal environments. Carbonate factories of the Wetterstein platform with microbially dominated ecosystems disappeared and shifted to less productive metazoan-dominated environments. Negative carbon isotope excursions (NCIEs) mirror the changes in the carbon precipitations. NCIE-1 marks the onset of the CPE in the Tethys Ocean and, at the Polzberg section, at the base of the Göstling formation at the transition from Julian 1 to Julian 2. Paleontological (ammonoids, bivalves, fish, conodonts, radiolaria, plants), lithological (thin sections, grey scale), geochemical (δ^13^C_carb_, δ^13^C_org,_ δ^34^S, δ^15^N, CaCO_3_, TOC, N, S), organic matter (OM) and hydrocarbons (n-alkanes, steroids, hypanoids), and geophysical data (magnetic susceptibility, gamma-ray) clearly show a transition from a carbonate to an argillaceous world during the formation of the Polzberg *Konservat-Lagerstätte* deposited during the onset of the globally warmer and more humid CPE. This indicates that the Reifling Basin and the adjacent Wetterstein platforms were affected by peculiar oceanographic conditions during this episode. The results highlight the complexity of the western Tethyan (Mediterranean) oceanography and special depositional basinal environments during prominent climatic perturbations. This study adds an important piece to our understanding of Carnian paleoceanographic history and climate. High-resolution studies on biostratigraphy, geochemistry and geophysics of the Polzberg section and the formation of the Polzberg *Konservat-Lagerstätte* from the NCA provide a reliable reference for future analyses of paleontological and geological issues.

The organic matter here is a mixture of aquatic and terrigenous material. The relative amount of aquatic biomass peaks in the topmost sample from the Göstling formation and the lower part of the Reingraben formation and decreases upsection. Bacterial sulfate reduction was an important process in organic matter degradation. Biomarker proxies indicate relatively high pH values during deposition of the upper Reingraben formation.

The section suffered a low thermal overprint as suggested by CAI and organic maturity parameters.

### Material and methods

#### Macrofossils

Samples were collected bed-by-bed, possible only in the lower calcareous parts, whereas in the upper argillaceous parts the sampling interval was 20 cm due to the soft nature of the deposits. Approx. 11,500 ammonoids were investigated in historical collections and collected during field campaigns in 2021 and 2022. Cephalopods dominate the nekton with ammonoids (72.2%) and coleoids (4.5%), followed by fish (6.0%). The macrobenthic community is dominated by countless halobiid bivalves. Additional less frequent members are other bivalves (1.0%), gastropods (1.6%), arthropods (3.0%), polychaetes (0.1%), coprolites and regurgitalites (10.1%) along with allochthonous plant remains (1.3%). The material is stored in the Natural History Museum in Vienna (NHMW, Austria).

#### Microfossils

A total of 52 thin-sections and 32 washed residue samples were made to study the microfacies and biostratigraphically important microfossils (conondontophorids, radiolaria, planktonic foraminifera). These data provide a high-resolution biostratigraphy, at least for the lowermost part of the section. Conodonts were investigated and photographed at the Department of Geoscience (University of Padova, Italy) and radiolaria at the Department of Physics and Geology (Università degli Studi di Perugia, Italy). Conodont and radiolaria specimens are stored in the Natural History Museum in Vienna (NHMW, Austria).

#### Lithology and facies

A total of 76 thin-sections were made to study the lithology, microfacies and biostratigraphically important microfossils (radiolaria, ostracods, planktonic foraminifera).

#### Geochemistry

Samples for CaCO_3_, TOC, S, and N content were collected with a spacing of 0.1–0.2 m and combined with grey-scale quantification. The samples were analyzed for total carbon (TC; %), total sulfur (TS; %) and total organic carbon (TOC; %) at the Chair Energy Geosciences (Montanuniversitaet Leoben; Austria) using an Eltra Helios CS elemental analyzer. TOC contents were measured on samples pre-treated with concentrated phosphoric acid. The accuracy of C and S measurements is better than 1%. Calcite equivalent percentages were derived using the equation calcite_equiv._ = 8.334 × (TC − TOC). The accuracy is better than 1%. In addition, calcium carbonate content was measured using a carbonate bomb technique^72^ at the Institute for Earth Sciences (Karl-Franzens-University, Graz, Austria). The accuracy is in the order of 2–3%^73^.

Main and trace elements were examined using the energy dispersive X-ray fluorescence (EDXRF) device Epsilon 5 (PANalytical). Each sample of 4 g, powdered < 60 µm, was mixed with 0.9 g wax (MERCK, Hoechst Wachs C Mikropulver) in a pebble mill Kugelmühle (Retsch MM200) and pelletized in a SPECAC press tool.

#### Rock–Eval data

Rock–Eval pyrolysis was performed at Montanuniversitaet Leoben (Austria) in duplicate using a Rock–Eval 6 instrument^74^. The determined parameters include the amounts of free hydrocarbons (S1 [mg HC/g rock]), hydrocarbons generated during thermal cracking (S2 [mg HC/g rock]), and the temperature of maximum hydrocarbon generation (T_max_ [°C]). S1 and S2 were used to calculate the hydrogen index (HI = 100 × S2/TOC) and the production index (PI = S1/[S1 + S2]^75^). The relative error for S2, HI and Tmax is about 2.0, 1.0 and 0.3%, respectively^74^.

#### Organic geochemistry

Eight samples were selected based on their TOC content and their stratigraphic position for biomarker analyses (Chair Energy Geosciences; Montanuniversität Leoben; Austria). 5 to 10 g of the sample material was extracted for about 1 h at 75 °C and 100 bar using dichloromethane as solvent and a Dionex ASE 350 Accelerated Solvent Extractor. The extract was concentrated to ⁓ 0.5 ml using a Zymark TurboVap 500 closed cell concentrator. Afterwards, asphalthenes were precipitated from a hexane: dichloromethane solution (80:1 according to volume) and separated by centrifugation. The hexane-soluble fractions were further separated into heterocompounds (NSO), saturated and aromatic hydrocarbons by medium-pressure liquid chromatography using a Köhnen-Willsch MPLC instrument^76^. Proportionate amounts of internal standards (*n*-tetracosane or squalene for aliphatics and 1,1ʹ-binaphthyl for aromatics) were added to each sample prior to measurement.

The *n*-alkanes and isoprenoids in the saturated hydrocarbon fractions were analyzed using a gas chromatograph (Trace GC-Ultra) with a flame ionization detector (GC-FID). The GC was equipped with a 50 m HP-PONA capillary column (inner diameter [i.d] 0.2 mm, 0.50 µm film thickness). After splitless sample injection at 270 °C, the oven temperature was increased from 70 to 310 °C followed by 35 min isothermal period.

Specific biomarker molecules in the saturated and aromatic fractions were analyzed by gas chromatography-mass spectrometry (GC–MS) using a Thermo Scientific Trace GC-Ultra equipped with a 60 m TG/DB-5MS fused silica capillary column (i.d. 0.25 mm; 0.25 µm film thickness) coupled to a ThermoFisher ISQ mass spectrometer. The GC oven temperature was initially programed from 40 °C/min, held for 2 min and ramped to 310 °C with 4 °C/min followed by an isothermal period of 40 min. The sample was injected in split mode with a split ratio of 20 at 260 °C using helium as carrier gas. The spectrometer was operated in the EI (electron ionization) mode with a mass-to-charge ratio (m/z) scan range from m/z 50 to m/z 650 (0.7 s total scan time). To determine methylsterane concentrations, we further investigated aliphatic fractions of selected samples using a Trace™ 1300 GC (ThermoFisher) equipped with a 60 m TG-5MS fused silica capillary column (i.e. 0.25 mm; 0.25 µm film thickness) attached to a ThermoFisher TSQ9000 triple quadrupole GC–MS/MS. The oven was set to hold 40 °C for 2 min and then heated to 310 °C with 4 °C/min, followed by a constant temperature period of 40 min. The sample was injected splitless with an injector temperature of 310 °C.

The raw data were processed using Xcalibur and Chromeleon data systems. The respective retention time for each compound within the mass spectra or total ion current (TIC) were used for their identification in addition to comparing the mass spectra with published data. Correction factors were appropriately applied for mass chromatograms to correct for fragment ions, whereas for TIC chromatograms, absolute concentrations were determined from the peak areas of the saturated and aromatic fractions in relation to their internal standards per sample.

A semi-quantitative calculation of the target compounds was conducted based on results of total ion current (TIC) chromatograms. Absolute concentrations were determined for the methylsteranes using their TIC chromatograms (also detectable using *m/z* 231) set in relation to an internal standard (5β(H)-Cholane) per sample.

#### Maceral analysis

Maceral analysis was performed on samples selected for biomarker analysis using white and fluorescence light, a 50 × oil immersion objective, and a Leica DM 4P microscope. At least 1000 points were counted, but given the generally low organic matter content, the results can be at best considered semi-quatitative.

The grey-scale data (0 = black, max. 255 = white, no dimension) indicate relative brightness intensity and were obtained by scanning 26 polished rock samples on a flatbed scanner (Epson Perfection 4990 Photo; method in Lukeneder et al.^66,77^).

A total of 32 samples were measured for bulk stable isotopes (δ^18^O, δ^13^C); additionally, δ^13^C_,_ δ^18^O and ^87^Sr/^86^Sr ratios of primary calcite were measured from a few ammonoid shells (original shell material aragonite) and one gastropod shell.

Isotopic analyses were performed in the Stable Isotope Laboratory at the Institute of Earth Sciences, Karl-Franzens-University, Graz, using an automatic Kiel II preparation line and a Finigan MAT Delta Plus mass spectromenter. Samples for the isotopic composition were drilled with a 0.3 mm-diameter dental drill. Samples were dried and reacted with 100% phosphoric acid at 70 °C: International standard NBS-18 and an internal laboratory standard were analysed continuously for accuracy control. The standard deviation was less than 0.08‰ δ^18^O and 0.04‰ for δ^13^C. Isotopic date are reported in conventional δ notation relative to the Vienna Pee Dee belemnite (V-PDB) standard in ‰ units and calculated with SMOW (standard mean ocean water). All isotopic data were measured at the Institute for Earth Sciences (Karl-Franzens-University, Graz, Austria) and are reported in conventional δ notation relative to the Vienna Pee Dee belemnite (VPDB) standard in ‰ units.

The ammonoid shell composition and internal microstructure were analysed at the laboratories of the Department of Material Sciences and Process Engineering (University of Natural Resources and Life Sciences, Vienna) by SEM imaging on a Quanta™ 250 FEG from FEI (environmental scanning electron microscope with a Shottky field emission source FEG-ESEM) with an EDS tool for microanalysis by energy-dispersive X-ray spectroscopy.

Low-field magnetic susceptibility (χ, MS) was measured directly in the field with a hand-held, highly sensitive (1 × 10^–7^, measured in SI units; results are indicated throughout as ‘value × 10^–6^ SI’), low-level SM-30 MS meter (GF Instruments) bearing a 50-mm pick-up coil. Bed-by-bed measurements were conducted at distinct beds (e.g. limestone or dolomite layers). This approach was replaced by a measuring interval of between 5 and 50 cm where distinct beds were missing (e.g. clay deposits, Reingraben formation); 112 measurements of MS were performed in sediments of the Reifling Formation (n = 12), Göstling formation (n = 10) and Reingraben formation (n = 90). Each single bed was measured at least three times and the average calculated. To prevent measurement errors due to the sensitivity of the hand-held instruments (uneven terrain and therefore variable distance between coil and rock surface), a fresh smooth surface was prepared for each single point measured.

#### Gamma-ray logs

Gamma response (gamma-ray logs) was measured in the field with 0.1–0.2 m spacing using a hand-held standard gamma-ray scintillometer (‘Compact Gamma Surveyor’, Gf Instruments). Deposits of the Reifling Formation (n = 12), Göstling formation (n = 10) and Reingraben formation (n = 72) were measured by 94 measurements.

### Supplementary Information

Supplementary Information.

## Data Availability

All sources of information are provided in the text. Raw data related to geochemistry and geophysics from Polzberg samples are available in supplementary data and from the corresponding author upon request. Images or additional information are available upon request from Alexander Lukeneder, Natural History Museum Vienna.
